# Prayers in Hospitals

**Published:** 1888-03-24

**Authors:** E. J. Waring


### PRAYERS IN HOSPITALS.

In your notice of my "Hospital Prayer-book" in your issue of February 25th there is a passage, based upon a misinterpretation, which I think likely to prove prejudicial to the interests of my book, and which therefore I should be very glad if you could see your way to correct in a future number. The passage in question is where I am made to say that the time occupied by the morning and evening prayers should be half-an-hour; whereas in fact none of the prayers occupy more than five minutes, and even these may be shortened at will, as may be seen by the passages in brackets, which may be omitted without impairing the sense of the prayers. The idea in my mind in speaking of half-an-hour was that that amount of time would be occupied in reading in two or more wards, and in the necessary passage to and from the hospital in the case of an outsider; not that the prayers themselves would take half-an-hour each, but that a person, by devoting half-an-hour to the work, might accomplish the "daily task" with ease and profit.

E. J. Waring.

[We regret having misunderstood Mr. Waring's intention, and are glad of this explanation, which confirms our conviction that short prayers are best in hospitals.]

